# Nitrogen fixation in a landrace of maize is supported by a mucilage-associated diazotrophic microbiota

**DOI:** 10.1371/journal.pbio.2006352

**Published:** 2018-08-07

**Authors:** Allen Van Deynze, Pablo Zamora, Pierre-Marc Delaux, Cristobal Heitmann, Dhileepkumar Jayaraman, Shanmugam Rajasekar, Danielle Graham, Junko Maeda, Donald Gibson, Kevin D. Schwartz, Alison M. Berry, Srijak Bhatnagar, Guillaume Jospin, Aaron Darling, Richard Jeannotte, Javier Lopez, Bart C. Weimer, Jonathan A. Eisen, Howard-Yana Shapiro, Jean-Michel Ané, Alan B. Bennett

**Affiliations:** 1 Department of Plant Sciences, University of California, Davis, California, United States of America; 2 Department of Agronomy, University of Wisconsin, Madison, Wisconsin, United States of America; 3 Department of Bacteriology, University of Wisconsin, Madison, Wisconsin, United States of America; 4 Genome Center, University of California, Davis, California, United States of America; 5 Department of Population Health and Reproduction, University of California, Davis, California, United States of America; 6 Instituto Tecnológico del Valle de Oaxaca, Oaxaca, Mexico; 7 Mars, Incorporated, McLean, Virginia, United States of America; University of Tübingen, Germany

## Abstract

Plants are associated with a complex microbiota that contributes to nutrient acquisition, plant growth, and plant defense. Nitrogen-fixing microbial associations are efficient and well characterized in legumes but are limited in cereals, including maize. We studied an indigenous landrace of maize grown in nitrogen-depleted soils in the Sierra Mixe region of Oaxaca, Mexico. This landrace is characterized by the extensive development of aerial roots that secrete a carbohydrate-rich mucilage. Analysis of the mucilage microbiota indicated that it was enriched in taxa for which many known species are diazotrophic, was enriched for homologs of genes encoding nitrogenase subunits, and harbored active nitrogenase activity as assessed by acetylene reduction and ^15^N_2_ incorporation assays. Field experiments in Sierra Mixe using ^15^N natural abundance or ^15^N-enrichment assessments over 5 years indicated that atmospheric nitrogen fixation contributed 29%–82% of the nitrogen nutrition of Sierra Mixe maize.

## Author summary

Nitrogen is an essential nutrient for plants, and for many nonlegume crops, the requirement for nitrogen is primarily met by the use of inorganic fertilizers. These fertilizers are produced from fossil fuel by energy-intensive processes that are estimated to use 1% to 2% of the total global energy supply and produce an equivalent share of greenhouse gases. Because maize (*Zea mays L*.) is a significant recipient of nitrogen fertilization, a research goal for decades has been to identify or engineer mechanisms for biological fixation of atmospheric nitrogen in association with this crop. We hypothesized that isolated indigenous landraces of maize grown using traditional practices with little or no fertilizer might have evolved strategies to improve plant performance under low-nitrogen nutrient conditions. Here, we show that for one such maize landrace grown in nitrogen-depleted fields near Oaxaca, Mexico, 29%–82% of the plant nitrogen is derived from atmospheric nitrogen. High levels of nitrogen fixation are supported, at least in part, by the abundant production of a sugar-rich mucilage associated with aerial roots that provides a home to a complex nitrogen-fixing microbiome.

## Introduction

Plants grow in close association with microbial communities that influence plant traits related to nutrient acquisition, plant development, plant defenses, and abiotic stress responses. The root-associated microbiota of plants has been characterized and shown to be much less complex than the microbiota of the surrounding soil, being enriched in Proteobacteria, Bacteroidetes, and Actinobacteria. These microbes are selected in part by plant cell wall features and metabolic cues from host cells [1,2]. Characterization of the rhizosphere microbiome associated with 27 modern maize (*Z*. *mays*) inbred lines also indicated substantial differences in relative abundance of microbial taxa between bulk soil and the rhizosphere, with the maize genotype contributing a small but significant influence on rhizosphere selectivity [3].

Nitrogen-fixing microbial associations with nonlegumes, especially cereals, have been a topic of intense interest for more than a century [4–7]. Nitrogen-fixing endophytes contribute to the nitrogen nutrition of sugarcane in some environments [8–10], but there is less evidence for the occurrence of efficient diazotrophic associations in other cereals. A 1-year study based on ^15^N dilution experiments in *Miscanthus × giganteus* suggested that this perennial bioenergy feedstock can acquire about 16% of its nitrogen from the air [11]. It has also been demonstrated that the model cereal, *Setaria viridis*, as well as *Setaria italica* (foxtail millet) can acquire a significant amount of fixed nitrogen from associations with *Azospirillum brasilense*[12,13]. Other examples of fixed atmospheric N_2_ being transferred to cereals include associations between *Azoarcus* sp. strain BH72 and Kallar grass [14], *Herbaspirillum seropedicae* and rice [15,16], and *Klebsiella pneumoniae* and wheat [17]. Because of its economic importance, the search for diazotrophic associations with maize (*Z*. *mays*) [18] has been a “holy grail” for decades, and several studies examined the contribution of nitrogen fixation by *H*. *seropedicae* and *Azospirillum* sp. to various maize accessions [19,20]. However, it is often difficult in these studies to distinguish the general plant growth–promoting benefits of these diazotrophic bacteria on yield from an actual transfer of fixed nitrogen to host plants. Five techniques are commonly used to evaluate nitrogen fixation: acetylene reduction assays (ARAs), ^15^N natural abundance, ^15^N enrichment, ^15^N_2_ gas enrichment, and nitrogen balance experiments [21,22]. All of these approaches have potential pitfalls, yet very few studies have compared different techniques or conducted assessments over multiple years to evaluate nitrogen fixation in nonlegumes.

Triplett suggested that it may be interesting to survey primitive maize landraces from the areas of maize origin to identify maize diazotrophic endophytes [5]. Estrada and colleagues [23] followed this suggestion and examined a landrace of maize in the Sierra Mixe region of Oaxaca, Mexico, and isolated a nitrogen-fixing endophyte from the resident maize landrace. The isolate was tentatively identified as a new species of *Burkholderia*, but the contribution of atmospheric dinitrogen (N_2_) to the nitrogen economy of the plant was not tested. This group also reported the isolation of a similar endophyte from field-grown teosinte plants and speculated that the *Burkholderia* strain might have formed a primitive symbiosis with teosinte that persisted during domestication of maize.

We also learned of isolated indigenous landraces of maize in the Sierra Mixe region of Oaxaca that were reportedly grown using traditional practices with little or no fertilizer and speculated that unique microbial community associations, not found in cultivated maize, might have evolved. This indigenous maize landrace is characterized by the extensive development of aerial roots that produce large amounts of mucilage. Mucilage associated with maize underground roots has been previously described [24,25], and it has been suggested that root exudates play a significant role in structuring rhizosphere microbial communities [26,27]. Indeed, it has been shown that pea root mucilage can serve as a sole carbon source for some rhizosphere bacteria, including *Rhizobium* sp., *Burkholderia* sp., and *Pseudomonas* sp. [28]. Aerial roots at the base of the maize shoot, also known as brace roots or nodal adventitious roots, can often reach the ground and are thought to provide anchorage to prevent lodging but may also contribute to nutrient and water uptake as well as gas exchange [29–31]. However, very little is known about the role of aerial roots that do not reach the ground and the mucilage that they produce. Here, we demonstrate that a Mexican maize landrace can acquire 29%–82% of its nitrogen from the air and that at least some of this N is fixed by diazotrophic bacteria present in the mucilage of aerial roots.

## Results

### Sierra Mixe maize morphology and mucilage

**Fig 1:**
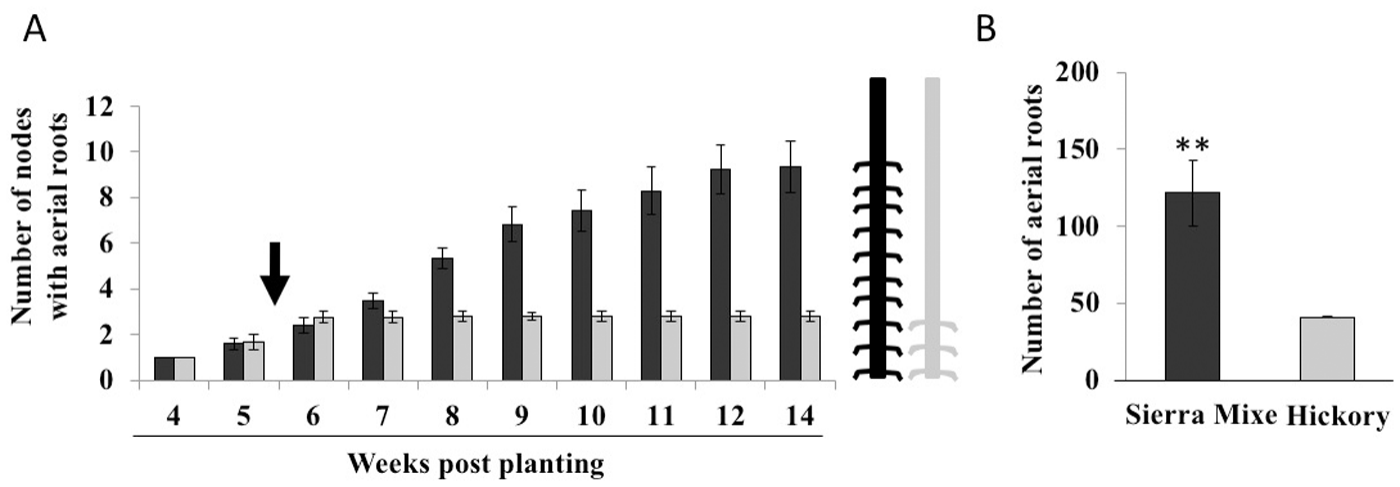
Physiological features of Sierra Mixe maize. (A) The transition between juvenile and adult phases (black arrow) occurs 5 weeks after planting in Sierra Mixe maize (black bars) and in the tall maize heirloom Hickory King (gray bars). (B) Number of aerial roots observed on Sierra Mixe maize and Hickory King after 14 weeks of growth in the field in Madison, United States of America. Error bars represent standard errors; an asterisk indicates a significant difference between Sierra Mixe maize and Hickory King (Student *t* test, *P* < 0.01). (Data at DOI: 10.6084/m9.figshare.6534545).

**Fig 2:**
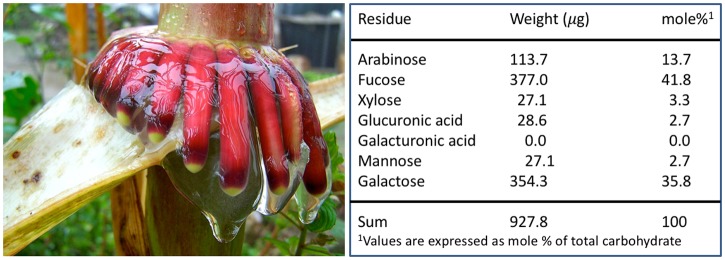
Aerial root mucilage. The aerial roots of Sierra Mixe maize (left) secrete large quantities of mucilage between 3 and 6 months after planting. The mucilage is carbohydrate rich, with the composition dominated by arabinose, fucose, and galactose (side panel).

The Sierra Mixe maize varieties cultured locally—referred to as Rojo, Piedra Blanca, and Llano—share similar plant morphologies, growing to a height of over 5 meters and exhibiting extensive aerial root formation at each node. We compared the development of aerial roots in the Sierra Mixe maize with another tall maize variety, Hickory King (Fig 1A). Unlike most modern maize varieties in which aerial root formation ceases after the juvenile-to-adult transition (arrow, Fig 1A), aerial root formation in Sierra Mixe maize continued well after this transition, resulting in a 3- to 4-fold greater number of aerial roots (Fig 1B). Approximately midway through development (July to September), these maize aerial roots secrete significant amounts of mucilage that is rich in arabinose, fucose, and galactose when moisture is available (Fig 2). The sugars comprise a complex polysaccharide that presumably contributes to the viscosity of the mucilage and may be disassembled to provide monosaccharides to support microbial growth and metabolism. Mucilage produced by underground maize roots also contain high levels of fucose and arabinose [32], although at approximately one-half the concentration found in aerial root mucilage.

### Sierra Mixe maize diazotrophic microbiota

**Fig 3:**
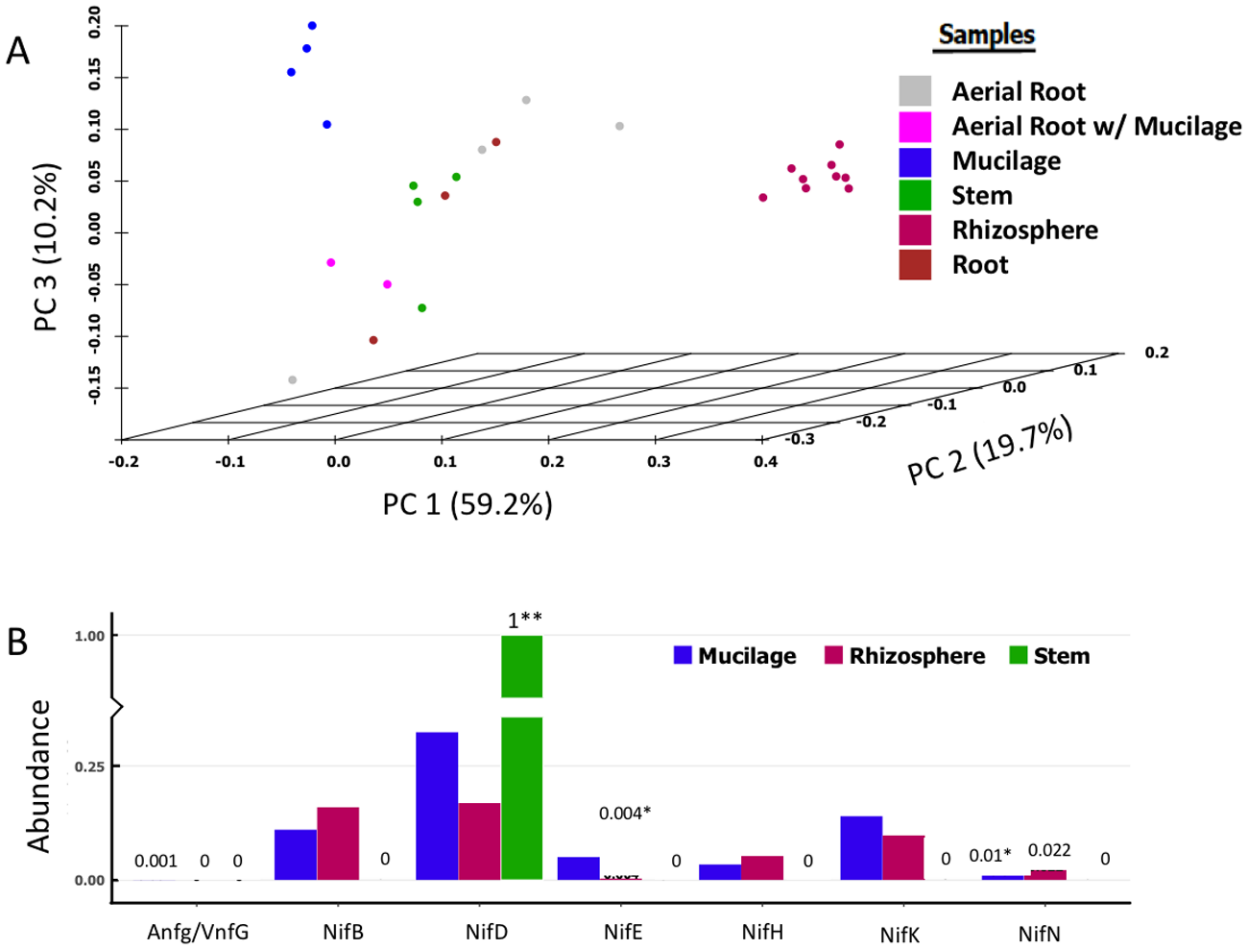
DNA sequencing–based characterization of the microbiome of Sierra Mixe maize. (A) Samples clustered by PCoA on Bray-Curtis dissimilarity distance matrix. Bacterial communities were assessed using PCR amplification and sequencing of rRNA genes. Each point corresponds to an individual sample. Permanova tests run using *adonis* in *vegan* revealed mucilage samples were statistically distinct (e.g., mucilage versus rhizosphere *P* = 0.002, mucilage versus roots *P* = 0.03, mucilage versus aerial roots *P* = 0.03). (B) Metagenomic sequencing–based analysis of homologs of core *nif* genes (*nifH*, *nifD*, *nifE*, *nifK*, *nifN*, and *nifB*) and alternate nitrogenase (*anfG*/*vnfG*). Metagenomic samples were searched for homologs by mapping reads on reference *nif* trees. The number of hits was normalized to an estimate of the number of bacterial genes in the metagenomic sample (measured using the number of hits to the RecA hidden Markov model). *The lowest abundance of a core *nif* gene in mucilage and rhizosphere libraries. **Only core *nif* gene hit in stem library. anfG and vnfG alternate nitrogenase were observed only in mucilage library. PCoA, principal component analysis.

The microbiota associated with the underground and aerial roots, stems, and aerial root mucilage of Sierra Mixe maize grown in Sierra Mixe was investigated by amplifying and sequencing of 16S rRNA genes and by shotgun metagenome sequencing. The rhizosphere samples were the most diverse, and among plant samples, the aerial root mucilage had the highest diversity (S1 Fig) and a higher relative abundance of bacteroidetes and proteobacteria (beta and gamma) compared to other parts of the plants (S2 Fig). Many of the lineages that are overrepresented in mucilage include known plant-associated nitrogen fixers. A comparison of samples based on the total community composition showed a clustering of mucilage samples that were statistically distinct from the rest of the plant and rhizosphere samples (Fig 3A). Clustering of samples based upon the variance-stabilized abundance of sequence variants again indicates that while the rhizosphere samples were distinct and diverse, the mucilage samples were distant from the other plant tissues (S3 Fig). The complete list of all sequence variants identified in the Sierra Mixe maize and soil samples can be found at (DOI: 10.6084/m9.figshare.4789759). Additionally, the metagenomic data was searched for homologs of the 6 core *nif* genes as described by Dos Santos and colleagues [33]. This search revealed the presence of all 6 core *nif* genes in the metagenomes from mucilage and rhizosphere and only a subset of the 6 core *nif* genes from stem tissue (Fig 3B). A higher normalized abundance of most *nif* genes (except *nifD*) in mucilage than stem tissues suggests that the mucilage may be enriched in nitrogen-fixing microbes.

**Fig 4:**
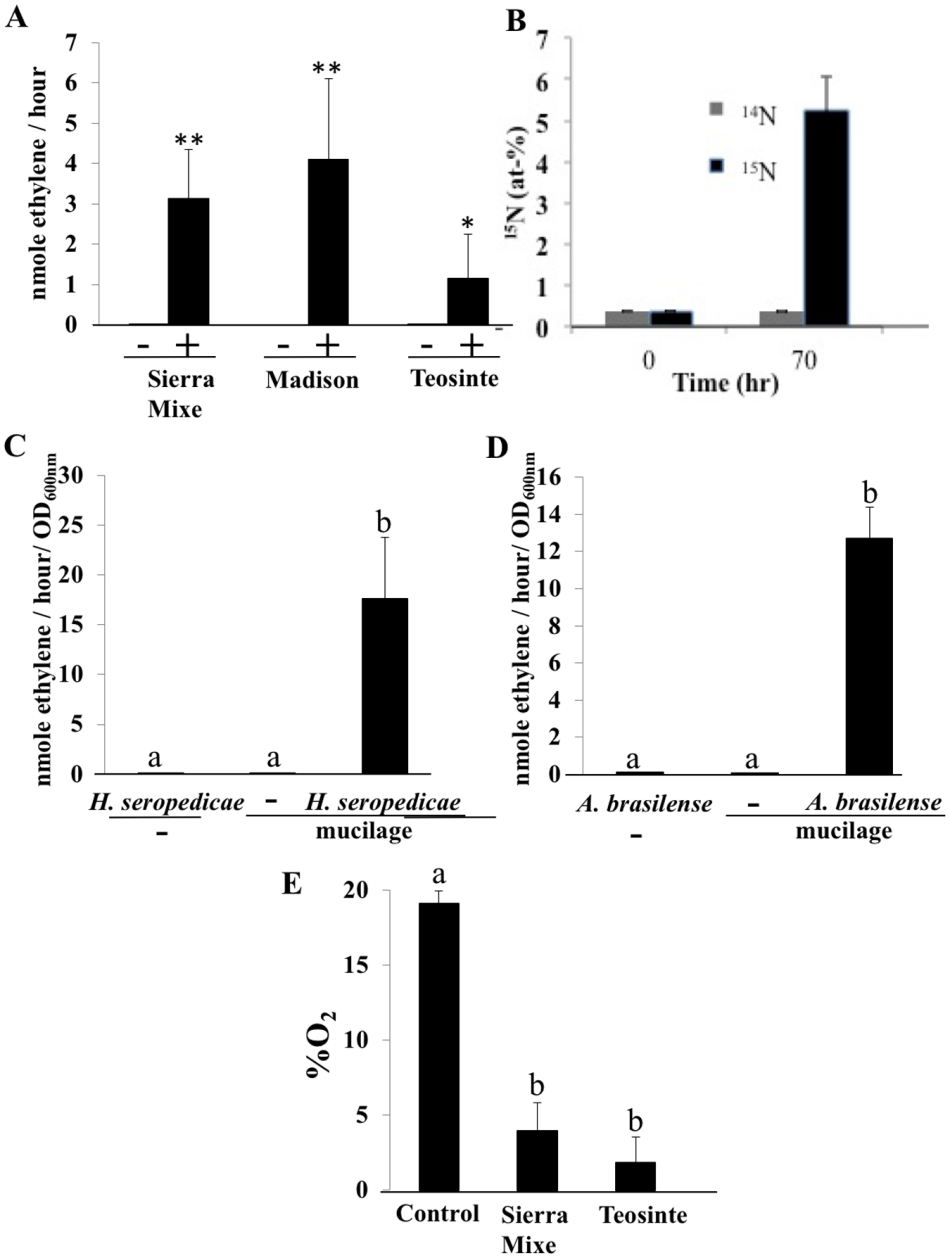
Nitrogenase and N_2_ fixation activity in mucilage produced by Sierra Mixe maize. (A) Mucilage of various Sierra Mixe maize lines collected in Sierra Mixe or field-grown plants in Madison, USA, and of teosinte display strong acetylene reduction activity. (-, no acetylene; +, 10% acetylene). Asterisks indicate significant differences (*P < 0.05; **P < 0.01, Mann-Whitney test). (B) Nitrogen fixation in Sierra Mixe maize mucilage by ^15^N_2_ assimilation. Mucilage collected from Sierra Mixe maize grown in Sierra Mixe was incubated in gas-tight vials filled with ^15^N_2_ or ^14^N_2_ gas for 70 hours at 37 °C. ^15^N (atom % excess) was determined by IRMS. (C and D) *H*. *seropedicae* and *A*. *brasilense* display acetylene reduction activity when added to nonfixing mucilage, whereas the same mucilage supplemented with sterile medium (-) or the same bacteria without mucilage (-) do not. (E) Oxygen concentration at 8 mm inside of the mucilage. Means and standard errors are shown. Different letters indicate statistically supported groups (Kruskal-Wallis test). (Data at DOI: 10.6084/m9.figshare.6534545). IRMS, isotope-ratio mass spectrometry.

To assess the possibility that mucilage harbored a diazotrophic microbial community, the mucilage was tested for nitrogenase activity using ARAs [34] and by incorporation of ^15^N_2_ gas. ARA was used to assess leaves, stems, underground roots, aerial roots (with and without mucilage), and mucilage collected from Sierra Mixe maize plants grown either in Sierra Mixe, Mexico, or Madison, USA. No ARA activity was detected in underground roots, leaves, stems, or even aerial roots before mucilage production (S4 Fig). In contrast, significant ARA activity was detected in aerial roots harboring mucilage (S4 Fig) and in isolated mucilage from plants grown in either Sierra Mixe or Madison (Fig 4A). The ARA activity in mucilage isolated from Sierra Mixe maize that was grown in Madison suggests either that the Sierra Mixe maize seeds carry an endogenous inoculum of nitrogen-fixing bacteria or that Sierra Mixe maize can recruit adequate nitrogen-fixing bacteria from local environments. Nitrogen fixation by mucilage samples was also measured by direct incorporation of ^15^N_2_ in mucilage samples collected from Sierra Mixe maize (Fig 4B).

As with Sierra Mixe maize, a wild relative, *Z*. *mays* ssp. *mexicana* (teosinte), also produced extensive aerial roots (S5 Fig) but much smaller amounts of secreted mucilage. To test the ability of the teosinte mucilage to support nitrogen fixation, we collected mucilage from several plants of teosinte and measured endogenous nitrogenase activity using ARA. Acetylene reduction was readily observed in teosinte mucilage (Fig 4A), suggesting that production of mucilage that supports nitrogen fixation by an associated nitrogen-fixing microbiota may be an ancient trait of maize and potentially introgressed from *Z*. *mays* ssp. *mexicana* into the Sierra Mixe landrace postdomestication.

To assess the mucilage characteristics that support nitrogen fixation, we tested the ability of 2 phylogenetically distinct nitrogen-fixing bacteria to reduce acetylene when inoculated in the mucilage collected from aerial roots of Sierra Mixe maize. Before the experiment, the mucilage was frozen for 2 weeks at –80 °C and thawed, which abolished endogenous nitrogenase activity (Fig 4C). Two nitrogen-fixing bacteria, *H*. *seropedicae*, and *A*. *brasilense*, showed readily detectable ARA activity when added to the mucilage (Fig 4C and 4D). Bacterial nitrogenase is O_2_-sensitive and needs to be protected by a low-oxygen (<5%) environment or physiological protective mechanisms, as well as an abundant carbon source to derive energy for this process [35]. To determine if mucilage could fulfill these requirements, we measured the free-oxygen concentration in the mucilage of Sierra Mixe maize and teosinte at the depth of 8 mm and found it to be <5%, indicating that the mucilage can provide a microaerobic environment compatible with nitrogen fixation for these bacteria [36] (Fig 4D). Mucilage is also comprised of complex sugars that may be catabolized to provide free sugars—mainly arabinose, fucose, and galactose—capable of supporting bacterial growth and nitrogen fixation. To determine whether these properties of the mucilage are sufficient to support nitrogen fixation, we created an artificial medium mimicking these mucilage properties by using a low-N medium, solidified with 0.2% agar, that reduced oxygen concentration to levels almost as low as those found in the mucilage (S6A Fig) and supplemented with a mix of free sugars corresponding to the composition of the fully hydrolyzed mucilage carbohydrates. *H*. *seropedicae*, *A*. *brasilense*, and *Burkholderia unamae* (S6B, S6C and S6D Fig) showed significant ARA activity in this reconstructed mucilage, indicating that the low O_2_ and free sugars provided by the aerial root mucilage are sufficient to support nitrogen fixation by these diazotrophs.

**Fig 5:**
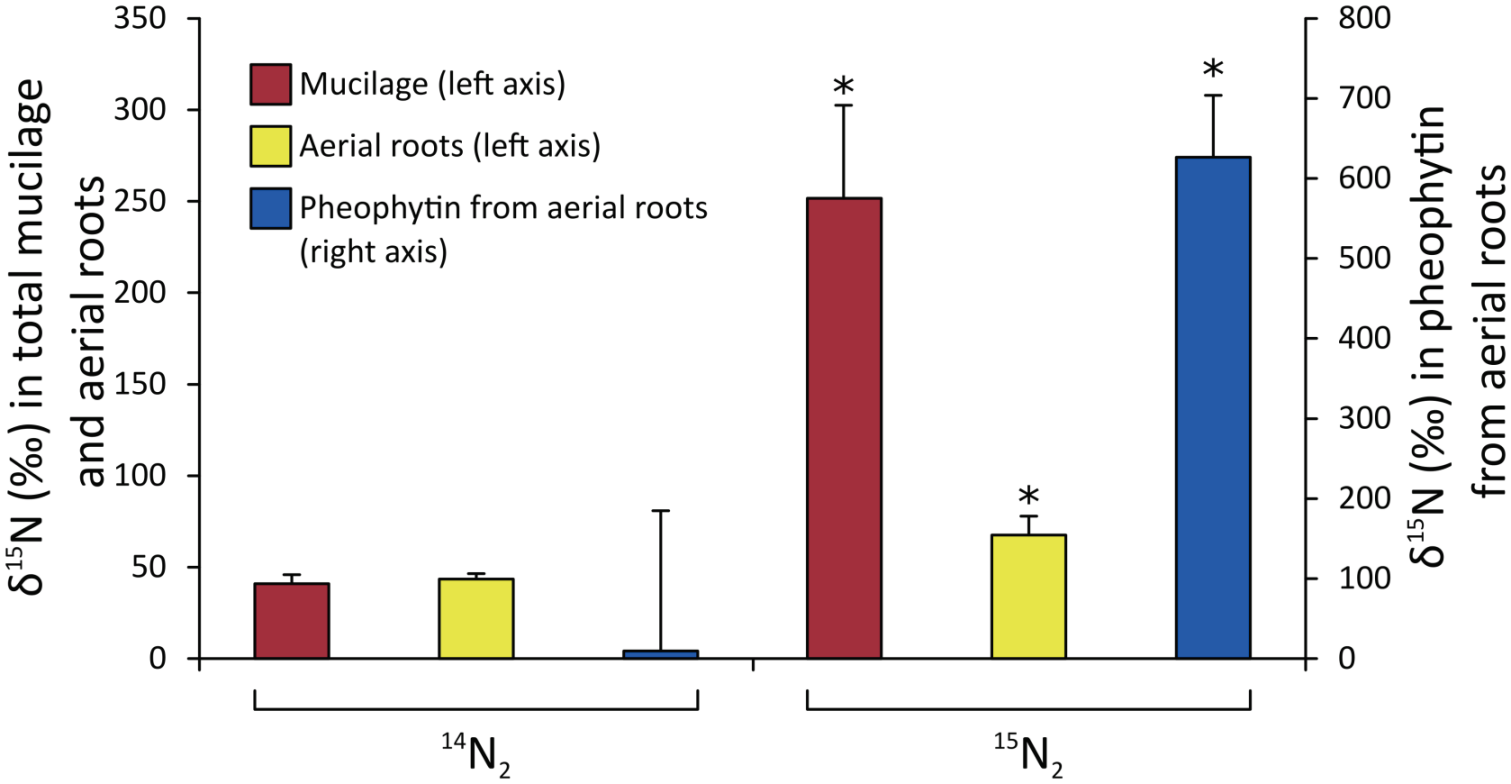
Analysis of Sierra Mixe maize samples for ^15^N_2_ enrichment in mucilage, aerial roots, and pheophytin from aerial roots. Mucilage was generated from aerial roots and inoculated with *A*. *brasilense* Sp7. ^15^N_2_ gas was pumped in and, after incubation mucilage, was separated from the aerial roots. Mucilage alone and aerial roots alone were subjected to analysis by IRMS. Results revealed a significant enrichment of ^15^N_2_ in mucilage alone and aerial roots alone (left y-axis). Since the inoculated aerial roots may contain *A*. *brasilense* Sp7 attached to the surface, we extracted pheophytin from these aerial roots to test ^15^N_2_ incorporation in pheophytin. Results revealed a significant enrichment of ^15^N_2_ in these aerial roots, indicating that aerial roots are indeed the sites for transfer of fixed nitrogen to the plants (right y-axis). ^14^N_2_ samples were used as negative controls. *n* = 4 (aerial roots and mucilage), *n* = 3 (pheophytin). The asterisk (*) indicates a statistically significant difference (*p* < 0.05). (Data at DOI: 10.6084/m9.figshare.6534545) IRMS, isotope-ratio mass spectrometry.

Based on ARA, we can conclude that mucilage from Sierra Mixe maize harbors native diazotrophs and can also support the N_2_-fixing activity of the exogenously inoculated diazotrophs, *H*. *seropedicae*, *A*. *brasilense*, and *B*. *unamae*. However, these data did not demonstrate that the aerial roots had the capacity to take up and assimilate the fixed N. To test whether atmospheric N_2_ that was fixed by mucilage-associated diazotrophs could be transferred to and utilized by the Sierra Mixe maize, a more direct ^15^N_2_ gas–enrichment experiment was used. Aerial roots, along with their generated mucilage, inoculated with *A*. *brasilense* Sp7 exhibited significant ^15^N_2_ gas incorporation in comparison to the ^14^N_2_ gas-treated roots (Fig 5), and isotope-ratio mass spectrometry (IRMS) analysis confirmed significant enrichment of ^15^N in chlorophyll (converted to pheophytin for analysis) of these roots compared to the negative controls (Fig 5).

### Nitrogen fixation contributes to maize N nutrition

The transfer of ^15^N_2_ from mucilage to the aerial root tissue and chlorophyll demonstrated the potential of this diazotrophic community to contribute to the nitrogen nutrition of the plant, but a major question of this study is whether the mucilage-associated diazotrophic microbiota served to transfer fixed nitrogen to fulfill, at least in part, the reduced nitrogen requirements of Sierra Mixe maize under field conditions. The contribution of atmospheric nitrogen fixation to Sierra Mixe maize was first estimated in the field using natural abundance ^15^N measurements [37,38]. This method relies on the relative abundance of the stable isotope ^15^N in the atmosphere and soil, with ^15^N abundance being more abundant in the soil than in the air. As a consequence, plants that derive N from the atmosphere will exhibit reduced δ^15^N levels when compared to reference nonfixing plants. In 2006, samples of Sierra Mixe maize and reference plants from the Asteraceae and Ranunculaceae (families with no known nitrogen-fixing members) growing near each other were collected from each of 2 fields. In this preliminary experiment, Sierra Mixe maize δ^15^N was significantly lower than the reference plants, indicating the assimilation of atmospheric nitrogen (S1 Table). In 2010, 2011, and 2012, the methods from [38,39] were used in experiments in Sierra Mixe by sampling reference species of non-nitrogen-fixing plants growing near the Sierra Mixe maize plants and a conventional maize variety, Maiz Blanco Conasupo. In addition to determining δ^15^N from each maize and reference plant sample, leaf samples of each reference plant were used for 18S rRNA sequence analysis to identify the reference species (Table 1A). The δ^15^N values of Sierra Mixe maize grown in the field in Sierra Mixe were determined at a single developmental time point in 2010 and at 5 developmental time points in 2011 and 2012. In 2010, the δ^15^N values for the Sierra Mixe maize were significantly lower than those of the reference plant species and of the conventional maize variety, Maiz Blanco Conasupo (Table 1A), indicating that the Sierra Mixe maize was able to derive a significant part of its tissue nitrogen from atmospheric dinitrogen. Similar δ^15^N values from root and leaf samples also showed that the leaf samples analyzed were representative of the whole plant (S2 Table). In 2011 and 2012, the δ^15^N values of Sierra Mixe maize were significantly lower than the reference plants at 4 of the 5 developmental time points, suggesting that Sierra Mixe maize derived a portion of its tissue nitrogen from atmospheric nitrogen (Table 1B). The calculated percent of nitrogen derived from the atmosphere (%Ndfa) from the δ^15^N values in Table 1 ranged between 30% and 80% (S7 Fig).

**Table 1:** Natural abundance ^15^N determinations. (A) δ^15^N values from Sierra Mixe maize, a conventional maize variety (Maiz Blanco Conasupo), and reference plants grown in Sierra Mixe, 3 months after planting. Values are given as mean and standard deviation. Statistical comparisons were made between the means of the reference plants, Maiz Blanco Conasupo, and *Z*. *mays* S. Mixe, using Student *t* tests (*p* < 0.05). Different letters indicate statistically supported groups. (B) δ^15^N values from Sierra Mixe maize plants grown in Fields 1 and 2 in Sierra Mixe during 2011 and 2012 at 2, 3, 4, 5, and 6 months after planting. Values are given as mean and standard deviation. Statistical comparisons were made between the reference plants mean and *Z*. *mays* S. Mixe means at each time point using Student *t* tests (*p* < 0.05). Different letters indicate statistically supported groups. Reference plants are listed in S3 Table. (Data at DOI: 10.6084/m9.figshare.6534545).

A		B		
Sample		Sample	2011 δ N15 (‰)	2012 δ N15 (‰)
*Rumex obtusifolius*	5.88 ± 0.44	Field 1		
*Ipomoea purpurea*	4.55 ± 029	Reference plants mean	5.86 ± 0.61 a	4.14 ± 0.35 a
*Bougainvillea* sp.	6.31 ± 0.49	*Z*. *mays* S. Mixe 2nd Month	4.57 ± 1.39 ab	6.38 ± 1.09 a
*Sambucus canadensis*	6.76 ± 0.09	*Z*. *mays* S. Mixe 3rd Month	2.36 ± 0.67 b	0.78 ± 1.26 c
*Hymenocallis chiapasiana*	4.08 ± 0.16	*Z*. *mays* S. Mixe 4th Month	3.92 ± 0.30 b	3.27 ± 1.98 b
*Physalis philadelphica*	6.12 ± 0.18	*Z*. *mays* S. Mixe 5th Month	4.04 ± 0.36 b	3.77 ± 0.33 b
*I*. *purpurea*	5.53 ± 0.20	*Z*. *mays* S. Mixe 6th Month	3.39 ± 0.20 b	2.66 ± 0.22 b
*Melampodiu perfoliatum*	4.29 ± 0.09			
**Reference plants mean**	**5.42 ± 0.21 a**	Field 2		
**Maiz Blanco Conasupo mean**	**4.87 ± 0.57 a**	Reference plants mean	5.19 ± 0.64 a	2.72 ± 0.26 a
*Z*. *mays* S. Mixe Field 1	3.05 ± 0.82	*Z*. *mays* S. Mixe 2nd Month	4.86 ± 1.39 a	−1.02 ± 0.90 bc
*Z*. *mays* S. Mixe Field 2	2.36 ± 0.94	*Z*. *mays* S. Mixe 3rd Month	2.10 ± 0.74 bc	−2.73 ± 1.07 c
***Z*. *mays* S. Mixe mean**	**2.71 ± 0.60 b**	*Zea mays* S. Mixe 4th Month	2.35 ± 0.52 b	−0.61 ± 0.23 b
		*Zea mays* S. Mixe 5th Month	1.20 ± 0.22 c	0.37 ± 0.31 b
		*Zea mays* S. Mixe 6th Month	2.74 ± 0.41 b	−0.41 ± 0.20 b

The method of using natural abundance ^15^N and other species as reference plants to calculate %Ndfa is potentially limiting because of differences in root and shoot growth and phenology of reference and test plants and the limited range of δ^15^N found in soils. An alternative method, ^15^N enrichment, is similar to the natural abundance methods but enriches soil ^15^N by the addition of ^15^N fertilizer, thereby increasing the difference between soil and atmospheric δ^15^N and assay sensitivity. Several direct comparisons have indicated that both methods can give comparable but slightly different results [38,40].

Another method of estimating N_2_ fixation is the “N Difference” method, which determines the difference between the total N content of an N_2_-fixing plant and the total N content of a reference nonfixing plant. Total N is calculated by multiplying total N content (%) in a specific plant sample and the total biomass (kg/ha or kg) produced by the plant. The %Ndiff is calculated as described in Materials and methods.

In 2016 and 2017, we assessed atmospheric nitrogen fixation using the ^15^N-isotope-enrichment method (1%–10% enrichment) at 3 vegetative growth stages—V9, V12, and Tassel—[41] in a random complete block design (5 replicates) in 3 low-N Sierra Mixe fields and at Tassel stage in 2017 in the same fields using the same design (Table 2). Field 3 (with a history of 0–1 year of maize production) yielded significant differences in Atom% ^15^N only for SM2 at the Tassel stage in 2016, but shoot N was significantly different in both 2016 and 2017. In 2016, Sierra Mixe maize landrace varieties exhibited significantly lower Atom%^15^N levels than the reference plants in Field 4, (with a history of 1–2 years of maize production) at Tassel and at V9 in 2016, and at Tassel in both years for shoot N. In both 2016 and 2017, Sierra Mixe maize landraces in Field 5 (over 3 years of continuous maize production) exhibited significantly lower Atom%^15^N and shoot N at all stages sampled, indicating a significant level of atmospheric nitrogen fixation in those experiments. The calculated %Ndfa ranged between 31% and 55%, and Ndiff ranged from 29%–82%. The correlation between Ndfa and Ndiff was 0.55 and 0.44 (*P* < 0.01) across locations in 2016 and 2017, respectively. Significant measures of N_2_ fixation were detected in 4 of 6 experiments by Atom% δ^15^N N determinations (Ndfa) and in 6 of 6 experiments by Ndiff determinations. Root and shoots exhibited significant differences in biomass, height, and stem diameter between control hybrids and local landraces (S4 Table). Soil analyses (S5 Table) showed that all sites were depleted in soil nitrogen yet produced a crop greater than 2,000 kg/ha. It is possible that differences between microbiota associated with the 3 fields in different stages of crop rotation (0 to >4 years of continuous maize production) account for differences observed among fields and between years, but further research is needed to answer this question.

**Table 2:** ^15^N-isotope-enrichment determinations in field trials. Percent Ndfa and Ndiff were calculated for Sierra Mixe varieties when Atom% ^15^N excess or Shoot N for the whole plant were significantly different from reference varieties as assessed by ANOVA and single-degree-of-freedom contrasts (*p* = 0.05), respectively. Values followed by asterisks are significantly different from the reference varieties based on single-degree-of-freedom contrasts (*p* < 0.05). Percent Ndfa and Ndiff were not calculated for reference (dashes). (Data at DOI: 10.6084/m9.figshare.6534545).

	2016 9-leaf				2016 12-leaf				2016 Tassel				2017 Tassel			
	Atom ^15^N (%)	Ndfa (%)	Shoot N (kg/ha)	Ndiff (%)	Atom ^15^N (%)	Ndfa (%)	Shoot N (kg/ha)	Ndiff (%)	Atom ^15^N (%)	Ndfa (%)	Shoot N (kg/ha)	Ndiff (%)	Atom ^15^N (%)	Ndfa (%)	Shoot N (kg/ha)	Ndiff (%)
**Field 3**																
Reference	0.60	-	5.1	-	0.28	-	21.3	-	0.21	-	12.0	-	0.24	-	5.9	-
SM1	0.35	43.5	11.1 *	46.4	0.19	30.2	31.7	29.1	0.18	21.5	27.3	46.5				
SM2	0.57	10.3	6.6	22.0	0.24	14.9	27.5	23.3	0.15*	29.0	34.2 *	60.6	0.23	5.0	24.2 *	75.0
**Field 4**																
Reference	0.16	-	21.8	-	0.12	-	58.5	-	0.09	-	58.2	-	0.21	-	28.5	-
SM1	0.17	5.6	31.5*	28.5	0.07	32.9	84.5	29.2	0.05 *	48.1	122.3 *	48.7	0.17	15.0	30.2	13.0
SM2	0.10	33.4	19.9	1.9	0.08	30.4	94.6	30.1	0.05 *	43.2	101.8	37.6	0.19	6.0	51.3 *	46.0
**Field 5**																
Reference	0.45	-	6.2	-	0.23	-	16.4	-	0.18	-	9.8	-	0.41	-	8.4	-
SM1	0.23 *	48.5	14.9 *	56.8	0.16	33.3	46.2 *	61.1	0.08 *	55.3	61.4 *	82.2	0.27 *	32.0	30.5 *	70.0
SM2	0.24 *	46.4	10.5	37.9	0.12 *	48.0	37.2 *	52.7	0.08 *	55.1	43.1 *	76.6	0.27 *	33.0	27.0 *	71.0

Abbreviation: Ndfa, nitrogen derived from the atmosphere; Ndiff, total nitrogen difference.

## Discussion

We have demonstrated that the mucilage associated with the aerial roots of Sierra Mixe maize can support a complex diazotrophic microbiota enriched for homologs of genes encoding nitrogenase subunits that harbor active nitrogenase activity, and that nitrogen is transferred efficiently from the nitrogen-fixing bacteria to the host plant tissues. Collectively, over several years and locations, the ^15^N natural abundance and ^15^N enrichment results of 2 selections of a Sierra Mixe indigenous maize landrace suggest that its nitrogen nutrition when grown in its native environment is partially fulfilled by fixation of atmospheric nitrogen. Nitrogen fixation is a particularly difficult phenotype to evaluate, as all the techniques available are prone to artifacts and can give different estimates for nitrogen fixation [42,43]. In this study, we used several classical techniques with Sierra Mixe maize and have shown over multiple locations and years that Sierra Mixe maize can fix nitrogen at rates (29%–82%) not previously reported, to our knowledge, in maize.

This study also revealed a new and important function for aerial roots and the mucilage they produce besides preventing lodging or water uptake [30]. This role in nitrogen fixation is probably the most important one for aerial roots that do not reach the ground. It will be interesting to explore if aerial roots produced by other cereals such as sorghum can perform a similar function [44]. We cannot rule out that diazotrophic activity in other parts of Sierra Mixe maize may also contribute to the acquisition of reduced nitrogen from the atmosphere. The developmental timing of the appearance of fixed atmospheric N_2_ in Sierra Mixe maize plants (Tables 1 and 2) before the extensive development of aerial roots suggests that there may, indeed, be additional sites of nitrogen fixation. However, we have not detected any significant nitrogenase activity outside of the aerial root mucilage.

The genetic basis of the trait or the source of the microbial inoculum, which may be either environmental or seed-borne, are unresolved. The observation that a teosinte species (*Z*. *mays* ssp. *mexicana*) also exhibits a similar diazotrophic activity in aerial root–associated mucilage suggests that this is an ancient trait that may have been introgressed and amplified in the Sierra Mixe landrace. It will be important, in the future, to determine the genetic basis of the trait, the identity of associated microbial diazotrophs, and the mechanisms of microbial recruitment. This research, together with other published research [5,18,23], suggests new avenues for research into potentially novel mechanisms of biological N_2_ fixation in maize. This could have a significant impact on maize crop productivity and nitrogen use efficiency, particularly in regions of the world where agriculture is characterized by poor soil nutrition.

## Materials and methods

### Plant material

Sierra Mixe maize seeds were obtained in Sierra Mixe region of Oaxaca, Mexico, from an open pollinated population. *Z*. *mays* ssp. *mexicana* (teosinte), LH123HT, Mo17, PHG39, LH82, PH207, and B73 seeds were obtained from USDA National Plant Germplasm System (NPGS) (accessions Ames 8083, PI601079, PI558532, PI600981, PI601170, PI601005, and PI550473, respectively). Maize line Hickory King was obtained from Victory Seeds (accession 3140041). Tornado-F21 and H-377 were obtained from Semillas Ceres in Oaxaca. SM1 and SM2 are different selections from the Sierra Mixe landrace. SM1 is a uniform population based on kernel size, shape, and color, and SM2 is a heterogeneous population representing the landrace.

Biological materials were accessed and utilized under an Access and Benefit Sharing Agreement between the Sierra Mixe community and BioN2, Inc., and with permission from the Mexican government. An internationally recognized certificate of compliance under the Nagoya Protocol (ABSCH-IRCC-MX-207343-3) has been issued for such activities.

### Bacterial strains and media

*A*. *brasilense* Sp7 and *B*. *unamae* MTI-641 were kindly provided by Dr. G. Alexandre (University of Tennessee, Knoxville, USA) and Dr. A. Hirsch (University of California, Los Angeles, USA), respectively. *H*. *seropedicae* Z152 (ATCC 35894) was provided by the ATCC (http://www.atcc.org/). Bacteria were grown in liquid BSE medium.

### Sample collection

The rhizosphere and plant tissues that include stem, leaf, aerial roots, underground roots, and mucilage of Sierra Mixe maize were sampled during seasons 2010, 2011, and 2012 from Fields 1 and 2 in Sierra Mixe. For plant endophyte analysis, tissues were surface sterilized by rinsing with mqH_2_O, shaken gently in 70% ethanol for 5 minutes, placed into 1% hypochlorite bleach, gently stirred for 10 minutes, rinsed 3 times in mqH_2_0, and dried in a laminar flow cabinet. Roots and stems were also dissected to remove epidermal tissues before extraction. For seed endophyte analysis, embryo and endosperm of Sierra Mixe, Hickory King, and B73 were withdrawn from the seeds by hand using a razor blade in a laminar flow cabinet and were collected in 1.5 ml sterile microcentrifuge tubes. For this study, the rhizosphere was defined as a layer of soil covering the outer surface of the root system that could be washed from roots in a buffer/detergent solution. Roots were first separated and shaken to remove loosely adhering soil. All soils and plant material samples were used immediately for DNA extraction. Soil fertility analysis, which included physical parameters, soil reaction, and salinity, was performed in AgroLab (Pachuma, Mexico).

### Plant phenotyping

The number of nodes with aerial roots was monitored weekly (greenhouse) or after 14 weeks (field). The total number of aerial roots was quantified after 14 weeks. The disappearance of leaf wax and appearance of trichomes were monitored weekly to determine the transition between juvenile stage and adult stage in Sierra Mixe and Hickory King maize.

### Greenhouse and field experiments, Madison, USA

For experiments in the greenhouse, seeds of Sierra Mixe of Fields 1 and 2 and Hickory King were surface sterilized and germinated as described previously. After 1 week, the seedlings are transplanted in 40-liter pots filled with a mix of sand and perlite (v:v) and grown in a high-ceiling greenhouse at the Biotron facility (University of Wisconsin, Madison, USA). Plants were watered twice a day for 2 minutes with half-strength of Hoagland solution. For experiments in the field, 3 independent plots of 20 plants per genotype were planted, with 3 border rows (B73) between each genotype. Sierra Mixe and Teosinte plants that were grown in Madison for the ARA were planted in the same field at the same time. This experiment was replicated in 3 different field plots.

### Mucilage glycosyl composition

Glycosyl composition analysis was performed by combined gas chromatography/mass spectrometry (GC/MS) of the per-*O*-trimethylsilyl (TMS) derivatives of the monosaccharide methyl glycosides produced from the sample by acidic methanolysis. Methyl glycosides were first prepared from dry mucilage samples by methanolysis in 1 M HCl in methanol at 80 °C (18–22 hours), followed by re-*N*-acetylation with pyridine and acetic anhydride in methanol (for detection of amino sugars). The samples were then per-*O*-trimethylsilylated by treatment with Tri-Sil (Pierce) at 80 °C (0.5 hours). GC/MS analysis of the TMS methyl glycosides was performed on an HP 6890 GC interfaced to a 5975b MSD, using an All Tech EC-1 fused silica capillary column (30 m × 0.25 mm ID). The analysis was performed at the Complex Carbohydrate Center Research Center (CCRC) of University of Georgia, Athens, USA.

### DNA extraction

DNA (80–150 ng μl^−1^) was extracted from 100 mg of the rhizosphere plant tissues using a DNA isolation kit (Mo Bio Laboratories, Carlsbad, USA). The PCR control for microbial DNA isolation was performed on 16S rRNA genes. PCR was performed using eubacterial primers 27F (5′-AGAGTTTGATCCTGGCTCAG-3′) and 1492R (5′-GGTTACCTTGTTACGACTT-3′), and the product was approximately a 1,450 bp fragment. Amplification was carried out with 1 μM of each primer in 3 mM MgCl_2_, 20 μM of each dNTP, 1.25 units of *Taq* polymerase (Promega) in a total volume of 20 μl of 1X reaction buffer (Promega, Madison, USA). PCR conditions included an initial denaturation at 95 °C for 3 minutes followed by 35 cycles of denaturation at 94 °C for 1 minute, annealing at 56 °C for 1 minute, and elongation at 72 °C for 1.5 minutes, with a final elongation at 72 °C for 7 minutes. DNA was resolved using an agarose gel run at 100 V for 30 minutes for analysis of total DNA and amplification of PCR products, respectively. Gels were visualized by ethidium bromide staining under UV light in a gel documentation system. The products obtained were purified with a NucleoSpin Gel extraction kit (Clontech, Palo Alto, USA).

### Illumina-based 16S rRNA gene sequencing

16S rRNA gene PCR and sequencing of rhizosphere and plant tissues were carried out using the Caporaso protocol [45]. We extended the Caporaso approach [46] to a dual barcode scheme for each sample and replaced the Golay barcodes with a different set of Illumina-compatible barcodes that were designed to balance base composition and tolerate up to 4 sequencing errors in barcode sequences. The forward primer used was AATGATACGGCGACCACCGAGATCTACAC[Barcode]TATGGTAATTGTGTGCCAGCMGCCGCGGTAA, and the reverse primer was CAAGCAGAAGACGGCATACGAGAT[Barcode]AGTCAGTCAGCCGGACTACHVGGGTWTCTAAT. The barcodes used were designed to allow pooling of multiple samples within a single MiSeq run. Ten cycles of PCR with barcoded primers were performed at low annealing temperature (55 °C); samples were then pooled and cleaned using a Qiagen column to remove the unincorporated primers. At this stage, an additional 10 or 20 cycles of PCR were performed on the pool using the Illumina paired-end flowcell primers with a higher annealing temperature (65 °C). The resulting PCR product was subjected to QC with an Agilent Bioanalyzer and estimated concentration using KAPA Biosystems qPCR kit. The samples were diluted to the appropriate loading concentration for a MiSeq run, spiked with 25% phiX control library, and sequenced using an Illumina MiSeq instrument with the manufacturer’s standard 150 nucleotides paired-end dual-index sequencing protocol and the custom sequencing primers.

The Illumina sequences were obtained from 2 MiSeq runs (2X150 bp paired-end) and were demultiplexed using a custom script (https://figshare.com/s/04997ae7f7d18b53174a). The 822,804 reads thus obtained were trimmed to a Phred-equivalent of 20 and filtered for adaptor contamination using BBDuk (of the BBTool packages https://sourceforge.net/projects/bbmap/). Because of the low quality of the reverse mate pair, reads of the forward mate were used in the analysis. The reads were then preprocessed and analyzed using DADA2 [47]. Prescribed standard filtering parameters were used, such as PhiX contamination check and removal of reads with more than 2 errors or ambiguous bases or with an expected error greater than 2. Chimeras were identified and removed using the removeBimeraDenovo function of DADA2. The clean reads were then collapsed into sequence variants and classified using RDP training set (version 14). The sequence variants that were classified as chloroplast or mitochondria were removed from further analyses. From samples with library size ranging from 84 to 20,597 reads (mean of 4,703), 995 unique sequence variants were identified.

Alpha and Beta diversity metrics were generated using the Phyloseq 3.4.2 R packages [48]. Alpha diversity was calculated using Shannon and Simpson indices. Additionally, a PCoA plot based upon Bray-Curtis dissimilarity matrix was used to visualize the differences in samples. The sequence variant table was used to generate a heat map following the variance-stabilizing transformation in DESeq2 [49]. Permanova tests were run using the *adonis* function from the *vegan* 3.4.3.R package (https://CRAN.R-project.org/package=vegan) performed on the NMDS ordination.

### Metagenomic sequencing

Illumina sequencing libraries from the same DNA extractions as above were made using an adaptation of the Nextera transposase-based library construction method with multiplex barcoding. Samples were then sequenced on the MiSeq and HiSeq instruments. Illumina sequences thus obtained were demultiplexed and trimmed using Trimmomatic (ver 0.33) [50] with the following parameter: Illuminaclip 2:30:10, Headcrop:15, Leading:20, Trailing:20, Sliding window:4:20, and Minlen:100. The reads were then screened for PhiX and maize sequences (genomic, chloroplast, and mitochondrial) using Bowtie2 aligner [51] against the PhiX genome (Genbank acc# NC_001422.1) and *Z*. *mays* cultivar B73 draft genome (RefSeq assembly acc# GCF_000005005.2). The clean reads were assigned taxonomy using Kaiju [52] with the nr database. To calculate the beta diversity of the samples, we used Phylosift (ver 1.0.1) [53], which identifies and places reads matching 37 conserved phylogenetic marker genes on a reference tree. From these placements, an Edge-PCA analysis [54] was carried using Guppy [54].

### *Nif* gene search

Peptide sequences of the 6 core *nif* genes (*nifH*, *nifD*, *nifE*, *nifK*, *nifN*, *nifB*) and alternate nitrogenase (*anfG*, *vnfG*) from known diazotrophs as previously published [33] were retrieved from GenPept as a reference. A multiple-sequence alignment of these sequences was generated as a reference alignment using ClustalW2 [55]. A blast search (E-value < 0.001) of 6 frame-translated metagenomic reads was conducted against these reference sequences. The hits were then aligned against the multiple sequences alignment of reference using clustal-Omega [56] followed by generation of phylogenetic trees for every individual *nif* gene, using Fasttree2.1 [57] with a WAG model of amino acid evolution and gamma20 likelihood. The reads were assigned as belonging to the *nif* genes if they were inside the clade of the reference sequences. Each read that had significant similarity to one of the core *nif* genes was further analyzed by phylogenetic analysis to confirm its assignment as one of the 6 core *nif* genes. The counts of nif genes thus obtained were normalized by *recA* counts (determined using *recA* TIGRFAM HMM [58] with HMMER3 and an E-value cutoff of e^−10^).

### Acetylene Reduction Assay ARA

For ARA with the mucilage, 2 ml of freshly collected mucilage from 1 or 2 aerial roots (Sierra Mixe) or several plants (teosinte) grown in the field were introduced in 14.5 ml vials (Wheaton, Millville, USA) that were tightly closed. For ARA with added bacteria, *A*. *brasilense*, *H*. *seropedicae*, and *B*. *unamae* were grown in BSE medium for 48 hours. Then, bacteria were collected by centrifugation (5 minutes, 4,000 × *g*) and suspended in the Fahraeus medium. The 14.5 ml vials, each containing 5 ml of mucilage previously stored for several months at –20 °C to reduce endogenous nitrogen-fixing bacteria, were inoculated with the bacterial suspension at a final OD_600nm_ = 0.01. Control tubes were prepared either without bacteria or with 5 ml of Fahraeus medium instead of mucilage. Then, 850 μl of acetylene (Airgas) was injected into each vial. OD_600nm_ was measured for each tube after 72 hours. For both conditions, controls without acetylene were performed in parallel. For ARA with aerial roots, 1 aerial root without mucilage was introduced in each 14.5 ml vial (10 replicates). One ml of acetylene (Airgas) was injected into each vial. For ARA with seedlings, Sierra Mixe seedlings were inoculated with *A*. *brasilense* and grown for 3 weeks. Plants were then transferred to 500 ml jars, and 50 ml of acetylene was injected in each jar. For ARA with underground roots, pieces of roots (about 10 cm long) were collected from plants grown in pots and introduced into 14.5 ml vials (3 replicates). Ethylene quantification was made by injecting 1 ml of the air phase, sampled after 72 hours, on a gas chromatography (GC-2010 Shimadzu) equipped with a Rt-Alumina BOND/KCL column (Restek).

### Mucilage ^15^N_2_ assimilation

The enrichment of mucilage in ^15^N atom was achieved by removing 4 ml of headspace gas and replacing it with 4 ml of either ^15^N_2_ (Sigma-Aldrich) or ^14^N_2_ nitrogen gas directly into a vial containing 1.0 mL of mucilage. Mucilage was collected from Sierra Mixe maize plants grown in Sierra Mixe and stored at 4 °C for up to 2 weeks between sampling and the determination of ^15^N_2_ assimilation. The mucilage samples were incubated at 37 °C for 0 and 70 hours in the presence of ^15^N_2_. ^15^N_2_ assimilation was stopped by freezing the mucilage samples at −20 °C. The samples were then freeze-dried and weighed. The ^15^N_2_ analysis in the mucilage samples was performed at the UC Davis Stable Isotope Facility (Davis, USA) and the UW-Madison Soil Science Facility (Madison, USA). Statistical analysis was performed using SYSTAT version 10 (Chicago, USA).

### Measurement of free-oxygen concentration

For measurement in collected mucilage, 2 ml of mucilage was introduced in a 15 ml tube. The probe (robust oxygen mini probe, Pyroscience) was introduced 8 mm deep in the mucilage and oxygen measurements performed until stabilization of the signal was observed. Control corresponds to free-oxygen concentration in the liquid Fahraeus medium. One-point calibration was made in aerated water, as advised by the manufacturer.

### ^15^N_2_ gas–enrichment experiments

Aerial roots were collected from Sierra Mixe maize grown at the Biotron greenhouse facility (University of Wisconsin, Madison, USA). Mucilage was generated from each of these aerial roots by incubating them in 5 ml of water at room temperature for 48 hours. Mucilage, along with the aerial roots, was inoculated with *A*. *brasilense* Sp7. Then, 10%–15% (v/v) of ^15^N_2_ gas was pumped into the vials, and the samples were incubated at 30 °C for 48 hours. After incubation of mucilage alone, or aerial roots alone, pheophytin extracted from these aerial roots was subjected to IRMS analysis. To obtain pheophytin, chlorophyll was extracted from aerial roots and converted to pheophytin by acid treatment, following as described [59]. ^14^N_2_-treated mucilage and aerial roots were used as negative controls.

### ^15^N natural abundance

The proportion (%) of nitrogen derived from biological nitrogen fixation (%Ndfa) was estimated from the ^15^N natural abundance (expressed in delta units, ‰) of the Sierra Mixe maize (δ^15^N_fixing plant_) and that of the reference plant species (δ^15^N_ref_). In each of the 2011 and 2012 field seasons in Sierra Mixe, 90–114 individual maize samples (depending on the year) and 270 reference plant samples, representing 8–10 species (depending on the year) of non-nitrogen-fixing plants, were analyzed. For the single time point in 2010, 12 individual maize samples and 33 reference plant samples, representing 8 species of non-nitrogen-fixing plants, were analyzed. The reference plant species in the field were identified using universal 18S PCR analysis from DNA sampled using FTA Plant Saver card (GE Life Sciences, Pittsburg, USA) simultaneously with tissue samples collected for ^15^N analysis. PCRs were performed using Sigma’s Extract-N-Amp Plant PCR Kit according to the manufacturer for sequencing and BLASTN comparison. For ^15^N natural abundance, the third-youngest leaf of Sierra Mixe maize or reference plants was collected from Field 3 and 4 from the second to the sixth month postplanting and analyzed for N-isotope composition. Total organic nitrogen was determined by Kjeldahl digestion followed by steam distillation. Analysis for natural ^15^N abundance was carried out as described by Bremer and van Kessel [38]. The ^15^N analysis was performed at UCD Stable Isotope Facility (http://stableisotopefacility.ucdavis.edu/13cand15n.html). The percentage of nitrogen derived from nitrogen fixation (%Ndfa) was calculated as follows:

$$\%Ndfa=\frac{\delta^{15}N_{\text{reference}}-\delta^{15}N_{\text{fixing}\;\text{plant}}}{\delta^{15}N_{\text{reference}}-\text{B}}X100,$$

where “δ^15^N” is stable nitrogen isotopes, “ref” is the value from non-N-fixing reference plants, “fixing plant” is Sierra Mixe maize, and “B” is the ^15^N abundance in the air, assumed to be 0.0‰.

### ^15^N-enrichment field experiments

In 2016, 3 locations were chosen: Field 3, land that had not been planted to crops for over 10 years; Field 4, land that had maize for 1 year; and Field 5, land with continuous maize. A randomized complete block design trial was established at each site, with 5 replicates with 4 varieties. Each plot consisted of 6 matas surrounded by a common border of SM2 on all sides and a double border on outside rows. A mata is the traditional planting design in the Sierra Mixe region, similar to a hill plot in which multiple plants are seeded together. Each mata was planted with 5 seeds and thinned to 3 seeds for 18 plants per plot. Matas were planted in a grid 80 × 100 cm from each other. ^15^N was applied at a dose of 0.36 grams per plot in a liquid solution, reaching the desired enrichment of 1% for all 3 fields, with 50 ml added per mata.

In 2017, the same 3 locations were planted with same design and entries, except Field 3 consisted only of H377 and SM2 entries. In all 3 fields, ^15^N was applied at a dose of 0.95 grams per plot, reaching the desired enrichment of greater than 1%. A solution was spread evenly over each plot using a garden watering can, such that the whole experimental area received an equal amount of enriched ^15^N. Plants were covered with plastic bags at the time of application (V5) to ensure that ^15^N was not directly applied to the leaves and that the ^15^N was uniformly available to all plants.

Soil samples were taken from a 0–60 cm depth in each plot, blended, and sent for analysis at UC Davis Soil lab. Means were calculated across locations. In 2016, at V9 and V12, 1 mata (3 plants) was sampled; and at Tassel, 4 matas (12 plants) were sampled. In 2017, a single sampling of 6 matas (18 plants) was sampled at Tassel. For each sampling, plants were dug out to include all roots. Because of the high rainfall (2,100 mm concentrated in the growing season from June to October), roots were shallow for both reference and test varieties. Each plant was photographed, and data were recorded for the number of plants, plant height, the total fresh weight of shoots, and roots and stem diameter. Whole plants were chopped, ground, subsampled, and dried in an oven to record total dry weight for shoots and roots. Well-blended subsamples were taken and shipped to Davis to measure Total N and ^15^N. Total nitrogen and Atom% ^15^N were determined for each plot at each time point for the shoot. Total organic nitrogen was determined by Kjeldahl digestion followed by steam distillation. The ^15^N analysis was performed at UCD Stable Isotope Facility (http://stableisotopefacility.ucdavis.edu/13cand15n.html).

%Ndfa was calculated using the ^15^N-enrichment method [40] as

$$\%Ndfa=(1-\frac{Atom{\%}^{15}N\;\text{excess}\;({\text{N}}_{2}\;\text{fixer})}{Atom{\%}^{15}N\;\text{excess}\;(\text{reference})})X100.$$

Atom% excess is calculated with sample value obtained from the UC Davis Stable Isotope Facility– 0.37 (n in air).

Nitrogen difference (%*NDiff*) method was calculated as

$$\%Ndiff=\frac{N\;kg/ha\;({\text{N}}_{2}\;\text{fixer})\;-\;\text{N}\;kg/ha\;(\text{reference})}{kgN/ha\;(\text{reference})}X100.$$

The average of Tornado F21 and H377 was used as the reference to calculate %Ndfa and %Ndiff. For Ndiff calculations, the area harvested was adjusted based on matas harvested at each time point.

### Field data analyses

Data were analyzed using the R lme4 package. Data were checked for outliers and subjected to ANOVA and mean separation using Least Significant Difference (*p* = 0.05) for each location. LSDs were calculated only when ANOVA F-tests were significant at *p* = 0.05. For %Ndfa and %Ndiff, single-degree-of-freedom contrasts were calculated to compare test varieties to the mean of the reference varieties (*P* > 0.05). Pearson correlation coefficients were calculated between %Ndfa and %Ndiff.

## Supporting information

S1 Fig Box plot indicating the alpha diversity as calculated by Phyloseq using (A) Simpson index and (B) Shannon index. (EPS)

S2 Fig Taxonomic distribution at the family level of the 25 most abundant bacterial families in (A) rRNA gene libraries and (B) whole-genome shotgun libraries. (EPS)

S3 Fig Heat map showing the hierarchal complete linkage clustering of samples. The heat map depicts the abundances of (A) 1,000 most abundant SVs and (B) 20 most abundant SVs in the dataset that were transformed using variance-stabilizing transformation in DESeq2. The libraries are Mucilage (Blue; OLMC00, OLMD00, OLMV00, OLMX00), Aerial Root (Gray; OLAR00, OLAR02, OLAR04, OLAR05), Aerial Root with Mucilage (Pink; OLAR01, OLAR03), Stem (Green; OLST00, OLST01, OLST02, OLST03), Underground Root (Brown; OLUR01, OLUR02, OLUR03), and Rhizosphere (Magenta; OXRZ11, OXRZ12, OXRZ13, OXRZ21, OXRZ22, OXRZ23, OXRZ32, OXRZ31, OXRZ33).
(EPS)

S4 Fig Nitrogenase (acetylene reduction) activity in different organs of Sierra Mixe maize. Significant nitrogenase activity was only found on aerial roots with mucilage.
(EPS)

S5 Fig Aerial root development in teosinte, Sierra Mixe maize, and a conventional variety, Hickory King grown in the field in Madison, USA. (A) Number of nodes with aerial roots and (B) number of aerial roots observed on teosinte, Sierra Mixe maize, and Hickory King after 14 weeks. Bar = standard error of the mean. Different letters indicate statistically supported groups according to the Kruskal-Wallis test.
(EPS)

S6 Fig Effect of reconstituted mucilage on oxygen diffusion and acetylene reduction. (A) Oxygen measured at 3 depths in Fahraeus medium with (black bars) or without (gray bars) 0.2% agar. (B) Effect of the different sugars present in the mucilage on the ability of *H*. *seropedicae*, (C) *A*. *brasilense*, and (D) *B*. *unamae* to reduce acetylene.
(EPS)

S7 Fig Proportion of nitrogen derived from biological N_2_ fixation (%Ndfa) in Sierra Mixe maize. Plants grown in Sierra Mixe during 2010 (light gray bars), 2011 (dark grey bars), and 2012 (black bars) were evaluated for %Ndfa; values were calculated using δ^15^N values in Table 1. Bar = standard error of the mean. %Ndfa, percent of nitrogen derived from the atmosphere.
(EPS)

S1 Table δ^15^N (‰) was determined in Sierra Mixe maize, and reference plants sampled from three 10 × 10 m locations were randomly selected from farmers’ fields in Sierra Mixe. From each location, 6 leaf samples were randomly sampled from Sierra Mixe maize plants and 6 leaf samples from each of 2 reference plants. The third emergent leaf of each maize plant was sampled. Reference plants were selected from the most abundant weed species within each sample location, and from a plant family (Asteraceae and Ranunculaceae) that is neither actinorhizal nor leguminous nor has members known to associate with diazotrophic bacteria. δ^15^N was determined for each plant sampled, and %Ndfa was calculated for Sierra Mixe maize according to the equation 2 in [19]. Values are given as mean and s.e. Different letters indicate statistically supported groups (one-way ANOVA, *P* < 0.05). %Ndfa, percent of nitrogen derived from the atmosphere.
(DOCX)

S2 Table δN^15^ (‰) distribution in root and shoot samples. Data are from a single sampling date (May 2012) in Sierra Mixe maize, with 30 replicates analyzed for each sample reported.
(DOCX)

S3 Table Reference plants sampled in the Fields 3 and 4 in Sierra Mixe in 2011 and 2012 and summarized in Table 2B. (DOCX)

S4 Table Shoot and root height and diameter measurements for field trials in Sierra Mixe in 2016 and 2017. Numbers followed by different letters are significantly different based on Least Significant difference at *p* = 0.05.
(DOCX)

S5 Table Soil analyses for samples taken at 0–60 cm before planting for fields in Sierra Mixe, Mexico. (A) Macroelements and soil characteristics. (B) Microelements for fields in 2017.
(DOCX)

## Abbreviations

%Ndfa: percent of nitrogen derived from the atmosphere
%Ndiff: percent total nitrogen difference
ARA: acetylene reduction assay
CCRC: Complex Carbohydrate Center Research Center
GC/MS: gas chromatography/mass spectrometry
IRMS: isotope-ratio mass spectrometry
NPGS: National Plant Germplasm System
PCoA: principal component analysis
TMS: trimethylsilyl
