# Supplementary material for: Nitrogen fixation in a landrace of maize is supported by a mucilage-associated diazotrophic microbiota
Source: PLoS Biol. 2018 Aug 7;16(8):e2006352. doi: 10.1371/journal.pbio.2006352 (PMC6080747; doi:10.1371/journal.pbio.2006352)
Supplement: S1 Table — From each location, 6 leaf samples were randomly sampled from Sierra Mixe maize plants and 6 leaf samples from each of 2 reference plants. The third emergent leaf of each maize plant was sampled. Reference plants were selected from the most abundant weed species within each sample location, and from a plant family (Asteraceae and Ranunculaceae) that is neither actinorhizal nor leguminous nor has members known to associate with diazotrophic bacteria. δ15N was determined for each plant sampled, and %Ndfa was calculated for Sierra Mixe maize according to the equation 2 in [19]. Values are given as mean and s.e. Different letters indicate statistically supported groups (one-way ANOVA, P < 0.05). %Ndfa, percent of nitrogen derived from the atmosphere. (DOCX) [file pbio.2006352.s008.docx]

| **Sample** | **δ N15 (‰)** |
| --- | --- |
| *Sierra Juarez maize* | 0.20 ± 0.60 a |
| Reference *Asteraceae* | 2.61 ± 0.46 b |
| Reference *Ranunculaceae* | 2.60 ± 0.20 b |
