# Supplementary material for: Nitrogen fixation in a landrace of maize is supported by a mucilage-associated diazotrophic microbiota
Source: PLoS Biol. 2018 Aug 7;16(8):e2006352. doi: 10.1371/journal.pbio.2006352 (PMC6080747; doi:10.1371/journal.pbio.2006352)
Supplement: S2 Table — Data are from a single sampling date (May 2012) in Sierra Mixe maize, with 30 replicates analyzed for each sample reported. (DOCX) [file pbio.2006352.s009.docx]

| **Species** | **δ^15^N (‰)**^+^ | **Total N (mg/kg)** ^+^ |
| --- | --- | --- |
| *Sierra Mixe* maize Shoot Field 1 | 4.14 ± 0.35 | 145.4 ± 39.4 |
| *Sierra Mixe* maize Root Field 2 | 3.08 ± 0.68 | 140.8 ± 43.7 |
|  |  |  |
| *Sierra Mixe* maize Shoot Field 1 | 2.72 ± 0.26 | 158.7 ± 54.8 |
| *Sierra Mixe* maize Root Field 2 | 2.36 ± 0.94 | 111.6 ± 34.4 |

^+^Mean values ± SE
