# Supplementary material for: Nitrogen fixation in a landrace of maize is supported by a mucilage-associated diazotrophic microbiota
Source: PLoS Biol. 2018 Aug 7;16(8):e2006352. doi: 10.1371/journal.pbio.2006352 (PMC6080747; doi:10.1371/journal.pbio.2006352)
Supplement: S3 Table — (DOCX) [file pbio.2006352.s010.docx]

| **2011** | **2012** |
| --- | --- |
| **Field 1** | **Field 1** |
| *Eleusine coracana* | *Lopezia racemosa* |
| *Smallanthus maculatus* | *Crusea calocephala* |
| *Lopezia racemosa* | *Trigonospermum annuum* |
| *Orchidantha chinensis* | *Lobelia laxiflora* |
| *Eleusine coracana* |  |
|  | **Field 2** |
| **Field 2** | *Lopezia racemosa* |
| *Lopezia racemosa* | *Echinopepon arachoideus* |
| *Rumex japonicus* | *Rumex nepalensis* |
| *Tropaeolum majus* | *Bidens aurea* |
| *Orchidantha chinensis* | *Ranunculus praemorsus* |
| *Amaranthus hybridus* | *Hydrocotyle verticillata* |
