# Supplementary material for: Nitrogen fixation in a landrace of maize is supported by a mucilage-associated diazotrophic microbiota
Source: PLoS Biol. 2018 Aug 7;16(8):e2006352. doi: 10.1371/journal.pbio.2006352 (PMC6080747; doi:10.1371/journal.pbio.2006352)
Supplement: S4 Table — Numbers followed by different letters are significantly different based on Least Significant difference at p = 0.05. (DOCX) [file pbio.2006352.s011.docx]

|  | **2016** | | | | **2017** | | | |
| --- | --- | --- | --- | --- | --- | --- | --- | --- |
| **Variety** | **Shoot (kg/ha)** | **Root (kg/ha)** | **Height  (cm)** | **Diameter  (cm)** | **Shoot (kg/ha)** | **Root (kg/ha)** | **Height (cm)** | **Diameter (cm)** |
| **Field 3** |  |  |  |  |  |  |  |  |
| Tornado-F21 | 745 bc | 82 b | 93.9 b | 1.82 |  |  |  |  |
| H-377 | 902 c | 53b | 83.1 b | 1.83 | 354 b | 15 b | 89 b | 0.98 b |
| SJ1 | 2343 ab | 216 a | 257.3 a | 1.72 |  |  |  |  |
| SJ2 | 2709 a | 329 a | 277.4 a | 2.06 | 2139 a | 200 a | 268 a | 1.75 a |
| **Field 4** |  |  |  |  |  |  |  |  |
| Tornado-F21 | 2465 c | 270 b | 140.2 b | 2.37 | 1494 b | 81 c | 151 b | 1.53 c |
| H-377 | 4144 b | 313 b | 139.7 b | 2.92 | 2014 b | 191 c | 156 b | 1.68 bc |
| SJ1 | 6898 a | 938 a | 351.1 a | 2.64 | 2607 b | 412 b | 307 a | 1.95 b |
| SJ2 | 5671 a | 851 a | 322.9 a | 2.79 | 4593 a | 840 a | 339 a | 2.40 a |
| **Field 5** |  |  |  |  |  |  |  |  |
| Tornado-F21 | 642 b | 109 b | 60.1 b | 1.27 a | 962 b | 45 b | 113 b | 1.12 b |
| H-377 | 638 b | 53 b | 80.7 b | 1.45 a | 315 b | 24 b | 86 b | 0.87 b |
| SJ1 | 4928 a | 443 a | 315.7 a | 2.44 b | 3024 a | 342 a | 304 a | 1.63 a |
| SJ2 | 3639 a | 391 a | 274.6 a | 2.32 b | 2765 a | 453 a | 314 a | 1.88 a |
