# Supplementary material for: Nitrogen fixation in a landrace of maize is supported by a mucilage-associated diazotrophic microbiota
Source: PLoS Biol. 2018 Aug 7;16(8):e2006352. doi: 10.1371/journal.pbio.2006352 (PMC6080747; doi:10.1371/journal.pbio.2006352)
Supplement: S5 Table — (A) Macroelements and soil characteristics. (B) Microelements for fields in 2017. (DOCX) [file pbio.2006352.s012.docx]

| **a)** | **pH** | **CEC meq/100g** | **OM (%)** | **NO3-N (ppm)** | **Olsen-P (ppm)** | **X-K (ppm)** |
| --- | --- | --- | --- | --- | --- | --- |
| Field 3 2016 |  |  |  | 18.78 |  |  |
| 2017 | 5.11 | 4.22 | 12.18 | 5.29 | 8.65 | 191.17 |
| Field 4 2016 |  |  |  | 51.10 |  |  |
| 2017 | 5.48 | 10.57 | 13.44 | 39.95 | 13.69 | 499.50 |
| Field 5 2016 |  |  |  | 17.11 |  |  |
| 2017 | 4.98 | 7.03 | 9.66 | 31.91 | 7.88 | 319.13 |

| **b) Field 2017** | **X-Na meq/100g** | **X-Ca meq/100g** | **X-Mg meq/100g** | **Zn (ppm)** | **Mn (ppm)** | **Cu (ppm)** | **Fe (ppm)** |
| --- | --- | --- | --- | --- | --- | --- | --- |
| Field 3 | 0.07 | 2.95 | 0.70 | 1.82 | 22.45 | 0.18 | 25.50 |
| Field 4 | 0.04 | 7.77 | 1.49 | 2.16 | 16.60 | 0.56 | 22.73 |
| Field 5 | 0.05 | 4.76 | 1.40 | 1.06 | 22.81 | 0.41 | 30.56 |
